# A New Method for Noninvasive Genetic Sampling of Saliva in Ecological Research

**DOI:** 10.1371/journal.pone.0139765

**Published:** 2015-10-23

**Authors:** Diana Lobo, Raquel Godinho, Francisco Álvares, José V. López-Bao, Alejandro Rodríguez

**Affiliations:** 1 CIBIO/InBIO, Centro de Investigação em Biodiversidade e Recursos Genéticos, Campus Agrário de Vairão, Universidade do Porto, Vairão, Portugal; 2 Departamento de Biologia, Faculdade de Ciências, Universidade do Porto, Porto, Portugal; 3 Research Unit of Biodiversity (UO/CSIC/PA), Oviedo University, Mieres, Spain; 4 Grimsö Wildlife Research Station, Department of Ecology, Swedish University of Agricultural Sciences (SLU), Riddarhyttan, Sweden; 5 Department of Conservation Biology, Estación Biológica de Doñana, CSIC, Sevilla, Spain; Smithsonian Conservation Biology Institute, UNITED STATES

## Abstract

Noninvasive samples for genetic analyses have become essential to address ecological questions. Popular noninvasive samples such as faeces contain degraded DNA which may compromise genotyping success. Saliva is an excellent alternative DNA source but scarcity of suitable collection methods makes its use anecdotal in field ecological studies. We develop a noninvasive method of collection that combines baits and porous materials able to capture saliva. We report its potential in optimal conditions, using confined dogs and collecting saliva early after deposition. DNA concentration in saliva extracts was generally high (mean 14 ng μl^-1^). We correctly identified individuals in 78% of samples conservatively using ten microsatellite loci, and 90% of samples using only eight loci. Consensus genotypes closely matched reference genotypes obtained from hair DNA (99% of identification successes and 91% of failures). Mean genotyping effort needed for identification using ten loci was 2.2 replicates. Genotyping errors occurred at a very low frequency (allelic dropout: 2.3%; false alleles: 1.5%). Individual identification success increased with duration of substrate handling inside dog’s mouth and the volume of saliva collected. Low identification success was associated with baits rich in DNA-oxidant polyphenols and DNA concentrations <1 ng μl^-1^. The procedure performed at least as well as other noninvasive methods, and could advantageously allow detection of socially low-ranked individuals underrepresented in sources of DNA that are involved in marking behaviour (faeces or urine). Once adapted and refined, there is promise for this technique to allow potentially high rates of individual identification in ecological field studies requiring noninvasive sampling of wild vertebrates.

## Introduction

The development of molecular techniques has increased the use of genetic analyses to test ecological hypotheses about the determinants of survival [1], habitat selection [2], dispersal [3], social organisation [4], demography [5], competition [6] or distribution [7]. Tissue samples as a source of DNA for these analyses can be advantageously replaced by noninvasive samples, especially for elusive animals [8]. For example, in mammals, faeces are the primary source of noninvasive DNA because they are easy to find, collect, handle and ship, and usually contain many epithelial cells [9,10]. However, faecal DNA is likely to be degraded to some extent, especially if faeces are not collected soon after deposition, which produces a quick decrease in amplification success and an increase in genotyping error rates over time [11,12]. Faeces also contain inhibitors of the PCR reaction [13,14]. The reduced quality of faecal DNA may pose problems for genotyping, especially when large molecular fragments are used [15], or the study population exhibits low genetic variability [16]. Therefore, obtaining DNA of high quality to establish the identity of individuals usually requires fresh faecal samples in the field and detection of many variable loci in the laboratory [17,18], conditions that greatly increase the cost of studies [10].

A further undervalued drawback of using faecal samples is that the researcher usually cannot manipulate their production by animals. The probability of finding a sample depends on the distribution, density and detectability of faeces, and possibly on the patterns of faecal marking. The distribution of resources and mates, social interactions, age, sex, status and other factors bring about individual differences in the number and frequency with which faeces are deposited in marking sites [19,20]. Moreover, faeces of marking individuals often appear either in aggregations or on prominent sites [19,20]. These conspicuous faeces may be more likely to be found than those released at random sites by individuals less or not involved in marking. Uneven probabilities of finding faeces produced by different individuals may limit the utility of faecal samples [21]. Indeed, by collecting only the most conspicuous faeces, a fraction of individuals in the population may remain undetected [17].

Saliva contains many cells and is an excellent source of DNA. In humans, the concentration of DNA extracted from saliva may be as high as, or higher than, that obtained from blood [22]. Saliva-based PCR can yield high levels of sensitivity and specificity [23]. Collection is simple and samples require no special storage [24]. Likewise in captive or wild vertebrates that can be easily restrained, saliva, other oral fluids or mucosae samples return similarly high DNA concentration and genotyping success [25,26].

Saliva DNA has been seldom used in settings requiring noninvasive sampling. Saliva is considered a suboptimal source of DNA compared with faeces or hair because suitable methods to systematically obtain saliva samples are lacking [10,27]. Noninvasive collection of saliva in the wild requires finding recently chewed plant food [28], recently killed prey [29,30], or any other fresh food remains. Collection of freshly deposited saliva seems unfeasible unless animals can be closely tracked to keep them in sight [28,31]. One alternative is luring target species to structures that will keep saliva after licking or chewing. Wax blocks that attract animals and can hold saliva traces have been designed to detect pest species through molecular methods [32]. More complex devices allow the selective collection of saliva samples [33], but this method is labour-intensive and human presence probably makes it inefficient for nocturnal or more wary taxa.

In this study we develop a novel noninvasive method to obtain saliva, based on a combination of edible lures and porous substrates that can absorb it. Substrates are designed to have a high probability to be expelled after bait consumption, and can thus be retrieved. The physical properties of the material used as substrate may influence the amount of saliva it can retain, whereas compounds found in baits may contain inhibitors of DNA amplification. Moreover, different bait-substrate combinations may induce variations in behaviours as handling and licking time which in turn may determine the amount of saliva produced, the amount of saliva stored in the substrate, and thus the probability of genotyping success. Therefore, in this study we test the performance of different combinations of baits and substrates by comparing 1) the amount of saliva attached to the substrate, 2) the concentration of DNA extracted from saliva, 3) species identification success, and 4) individual identification success. We also quantify genotyping errors and the effort needed to achieve individual identification in terms of number of replicates. Finally, we examine whether the number of replicates needed for successful genotyping was related with the quantity of saliva collected and DNA concentration in extracts. Differences in relative performance may help detect issues that should be considered when adapting the substrate-bait technique of saliva collection to specific field studies.

## Materials and Methods

### Sampling Procedure

To explore the potential of this method we designed a test in controlled conditions using captive dogs as experimental subjects. Four experimental sessions were conducted in a charity kennel between November 2012 and January 2013. A fifth session was carried out at the kennel of the Institute of Biomedical Sciences Abel Salazar (University of Porto) in June 2013. Dogs of both sexes (45% females; n = 29), varying sizes (range = 8–29 kg) and breeds (83% were mongrels) were kept in paved, indoor individual pens, except for experimental session 2 which had to be carried out in unpaved pens. Trials took place at 09:00, before dogs were fed.

**Fig 1:**
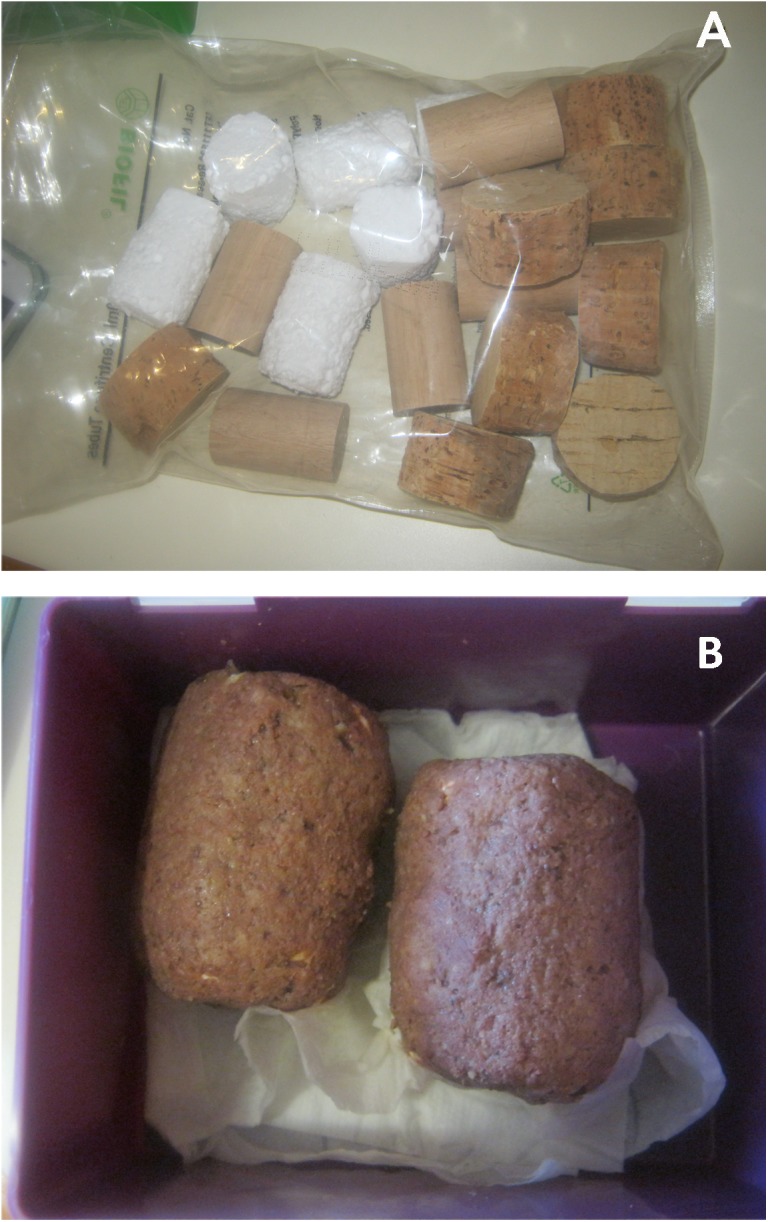
A: Porous substrates (cork, styrofoam, wood) in a bag after sterilization. B: Cork substrates covered with meat, ready for delivery.

As a substrate for saliva collection we chose three materials with different porous surfaces that are cheap and commonly found (Fig 1). We prepared cylinders of raw cork (⊘ = 6 cm; length = 3.5 cm), expanded polystyrene foam (henceforth styrofoam; ⊘ = 4 cm; length = 5.5 cm) and wood (⊘ = 3.5 cm; length = 5.5 cm). We removed any traces of DNA by sterilizing substrates with ultraviolet light for a period of 30 min and then we kept them in sterile plastic bags. To make substrates appealing to dogs we covered them with a 5 mm layer of soft bait just before delivery (Fig 1). We used two types of bait, either canned meat for dogs (henceforth meat) or a paste resulting from mixing canned sardines in vegetable oil (sardines). Commercial food brands were kept the same throughout the study. Cost of these saliva traps did not exceed 0.3 € per unit. We delivered covered substrates by tossing them on the ground.

In each experimental session, observations were made on six dogs, except for session 3 in which we excluded partial data from one individual that was adopted during the trial. During a nine-day period each dog was exposed once to each of the six possible combinations of substrate and bait. To avoid the potential effect of habituation, supply was interrupted twice in each session. Habituation could make some individuals lose interest in substrates during the late days of the session. This uneven attraction could result in lower consumption rates or a decreased probability of collecting saliva, and eventually could unbalance sample sizes across treatments. Therefore for each dog we delivered a single substrate-bait combination on days 1, 2, 4, 5, 8 and 9. The sequential assignment of substrate-bait pairs to dogs was random. Each individual was used in only one session.

Dogs usually ate the bait and then chewed or licked the substrate. This behaviour was videotaped. In videos we measured how long was the substrate inside the dog’s mouth (chewing time), and how long was the substrate licked (licking time), being handling time the sum of both values. Once dogs left the substrate unattended for 1 min we collected it using sterile gloves. We scored the quantity of saliva attached to the substrate from 1 to 4 with a step of 0.5, where value 1 was assigned to apparently dry surfaces and value 4 to surfaces soaked with saliva. We called this semi-quantitative variable “saliva score”. Score 4 roughly corresponded to a 1 mm-thick layer of saliva covering the whole surface, which would give a maximum volume of 8–12 ml depending on the substrate. Then we stored the substrate in 96% ethanol until DNA extraction. Before the outset of each session we plucked about 10 hairs from each experimental dog and stored them in 96% ethanol as an independent source of DNA for individual identification.

## Laboratory Procedures

We extracted DNA only from samples obtained in the first four sessions of the experiment. Substrates were first removed from ethanol and dried at 37°C, and were then vigorously washed with 15 ml of PBS solution to release cells into the solution. Cells were recovered from PBS with a centrifugation step of 25 min at 4000 rpm and were resuspended in 300 μl of lysis buffer from the commercial kit QlAamp DNA Micro Kit (QIAGEN, Hilden, Germany). DNA extraction followed then the manufacturer’s instructions for that kit. A maximum of 14 samples and one negative control to monitor potential contamination were handled in each extraction round. For each dog, DNA was extracted from the pooled set of sampled hairs. DNA extraction from hair samples also followed the QIAamp DNA Micro Kit protocol with a pre-digestion step of 12 h at 56°C. DNA was eluted in 50 μl of elution buffer.

DNA concentration in extracts of saliva samples was measured through fluorescence using a Wallac Victor fluorometer (PerkinElmer, Waltham, MA, USA) set at 480 nm for excitation and 520 nm for emission according to the instructions of the Quant-it PicoGreen dsDNA Assay Kit (Life Technologies, Carlsbad, CA, USA). The average value of three concentration measurements was assigned to each sample. DNA was diluted to 10 ng μl^-1^ when sample DNA concentration was above this threshold. Otherwise, no dilution/concentration was applied.

As our approach was designed for use with DNA of uncertain origin collected in the field, we explored how well our method was able to determine species identity from DNA in saliva extracts. To perform species identification, a 425-bp fragment of the mitochondrial (mtDNA) control region was amplified using universal primers Thr-L 15926 and DL-H 16340 [34]. mtDNA amplification was prepared in an 11 μl final volume reaction containing 2 μl of DNA and using the Multiplex PCR Kit (QIAGEN) following PCR conditions given by the manufacturer and with the annealing temperature (*Ta*) set to 52°C during 40 cycles. Successful amplifications were purified using enzymes exonuclease I and Shrimp alkaline phosphatase and sequenced with BigDye chemistry (Applied Biosystems, Foster City, CA, USA) using the same primers. Sequencing products were separated on an ABI 3130xl Genetic analyzer (Applied Biosystems). Electropherograms were aligned and checked using SEQSCAPE 2.5 (Applied Biosystems). Only one mtDNA amplification per sample was performed.

Individual multilocus genotypes were determined using a set of ten dog autosomal microsatellites (see S1 Table for loci description, primer concentration, dye and references). All loci were amplified in a single multiplex reaction using the Multiplex PCR Kit and a primers mix previously prepared (S1 Table) in a 10 μl final volume containing 2 μl of DNA. Fluorescent labelling of PCR fragments was accomplished following Blacket et al. [35]. Thermocycling was performed in a MyCycler Thermal Cycler (Bio-Rad, Hercules, CA, USA) using a touchdown profile with the *Ta* decreasing 0.5°C per cycle, from 60°C to 57°C in seven cycles, followed by 22 cycles with *Ta* set to 57°C and eight more cycles with *Ta* set to 53°C. We performed three replicates per amplification using the multi-tubes approach [36,37]. Hair samples were genotyped using the same procedure without replicates. All reactions were monitored for possible contaminants using negative controls. PCR products were separated by size on the same genetic analyzer using the 350ROX size standard. Alleles were determined using GENEMAPPER 4.0 (Applied Biosystems) and checked manually.

## Individual Identification

Reference individual identities were obtained from hair sample genotypes. For each replicate of each saliva sample we compared the genotype observed at each locus with the reference genotype. Identification failure occurred when we observed a mismatch or no amplification, so individual identification success was a binary variable. Moreover, we calculated the proportion of loci with the correct genotype by taking the highest value out of three replicates for each sample. We determined the rate of allelic dropout and false alleles for each of the ten loci using GIMLET 1.3.3 [38].

As lab costs increase with the number of genotyping replicates, we analyzed whether the number of replicates required for individual identification varied with types of substrate and bait. We compared the three replicates from each sample with the corresponding hair sample and evaluated the number of replicates needed to achieve a complete genotype. Values of probability of identity (*PI*) and probability of identity among siblings (*PI*_sibs_) were calculated in GIMLET as 1.86 10^−10^ and 1.34 10^−4^, respectively. Number of replicates varied in the range 1–4, where value 4 indicated that three replicates were not enough for individual identification.

Finally, we used the three genotyping replicates to calculate individual identification success without direct comparison to the reference genotype. This is relevant because field studies using our method will not always have access to control DNA samples. To determine the consensus genotype of each sample we first estimated allelic dropout and false alleles rates using a maximum likelihood approach implemented in the PEDANT software [39]. Next we estimated the minimum number of repetitions that confirms an allele per locus (consensus threshold) using 1000 simulations, as implemented in GEMINI 1.3.0 [40]. Then, using the consensus threshold we estimated the consensus genotype for each sample using GIMLET. Finally, the consensus genotype was used as a reference against which to determine individual identification success of saliva samples expressed as the percentage of matching loci in each replicate.

## Statistical Analyses

As DNA was extracted only in samples from dogs in the charity kennel, data from the first four experimental sessions were used in subsequent analyses. Given the factorial design of the experiment, we examined the null hypothesis that all kinds of substrate were able to store the same amount of saliva regardless the type of bait used. Using saliva scores as the response variable, we fitted a Generalized Linear Mixed Model (GLMM) with normal errors containing predictors of interest and confounding variables as fixed factors, as well as codes for experimental session and individual identity as random factors. Dog identity was nested within experimental session. Fixed predictors of interest included substrate, bait, their interaction, as well as chewing, licking and handling times because longer handling of a substrate either inside dog’s mouth or while licking it on the ground could increase the amount of deposited saliva. Handling time was positively correlated with its two components, but chewing time and licking time were uncorrelated (*r* = 0.087, *P* = 0.379). As a result, any pair of these three variables was allowed to be a part of competing models but the three predictors were not entered together in the same model. As saliva production might vary with dog sex, dog size and habituation (expressed by exposure time), these variables were included in the model to account for their potentially confounding effect. We categorized dog size as small (weight <15 kg) or large (≥15 kg). We measured exposure time in days since the beginning of the experimental session. We subjected the full GLMM to backward stepwise simplification by removing non-significant effects and using fit statistics to compare the parsimony of simplified models [41]. For normally distributed response variables, we used Tukey contrasts to compare means across substrate types.

We applied the same analytical approach to other response variables after the necessary modifications. In the analysis of DNA concentration in extracts, we included saliva score in the models as a fixed effect, because a large volume of saliva may contain more cells and thus more DNA. When modelling the success of species and individual identification, either as a binary variable expressing a fully matching genotype or a binomial variable expressing the proportion of loci correctly identified in reference and consensus genotypes, we used binomial errors and included the amount of saliva attached to substrates and the concentration of DNA in saliva extracts as predictors in the full model. Likewise these predictors were considered in models with Poisson errors to analyze the number of genotyping replicates needed to reach a correct individual identification. Log pseudo-likelihood methods of estimation in models with binomial and Poisson errors precluded the use of fit statistics as criteria for model simplification [42].

### Ethics Statement

This research complied with legal requirements in animal experimentation and was approved by the University of Porto Ethics Committee (permit number 026/2012). Experimental procedures did not produce any animal suffering.

## Results

Overall we supplied substrates to 29 individuals in 174 occasions. We retrieved the substrate in 98% occasions, as dogs destroyed styrofoam substrates four times. We assessed the amount of saliva in all substrates and performed genetic analyses on 134 pieces handled by 23 different dogs, for which we obtained hair samples. In this sample, mean (±SE) handling time was 61.6±3.7 s (*n* = 133) of which 40% was allocated to chewing (25.0±2.7 s; *n* = 129) and 60% to licking (36.8±2.6 s; *n* = 129). For wood substrates, mean chewing and handling times were significantly longer than for other substrates (Table 1).

**Table 1:** Variation in dog handling behaviour across types of substrates and baits. For substrates, Tukey contrasts for pairs of means were indicated by shared superscripts: ^a^ *P*<0.001, ^b^ *P* = 0.023, ^c^ *P* = 0.084, ^d^ *P* = 0.002.

		Chewing time (s)				Licking time (s)				Handling time (s)			
	*n*	Mean	*SE*	*F*	*P*	Mean	*SE*	*F*	*P*	Mean	*SE*	*F*	*P*
Substrate													
Styrofoam	40	10.4^a,c^	1.36			34.7	4.34			46.2^d^	4.14		
Cork	44	23.5^b,c^	4.15			37.1	4.33			61.1	5.60		
Wood	45	39.4^a,b^	5.75			38.3	4.76			76.6^d^	8.12		
				11.201	<0.001			0.167	0.846			5.817	0.004
	*n*	Mean	*SE*	*t*	*P*	Mean	*SE*	*t*	*P*	Mean	*SE*	*t*	*P*
Bait													
Meat	65	27.4	4.38			36.8	3.51			64.1	5.75		
Sardines	65	22.5	3.05			36.7	3.78			59.1	4.92		
				0.901	0.369			0.034	0.973			0.661	0.510

### Amount of Saliva

**Fig 2:**
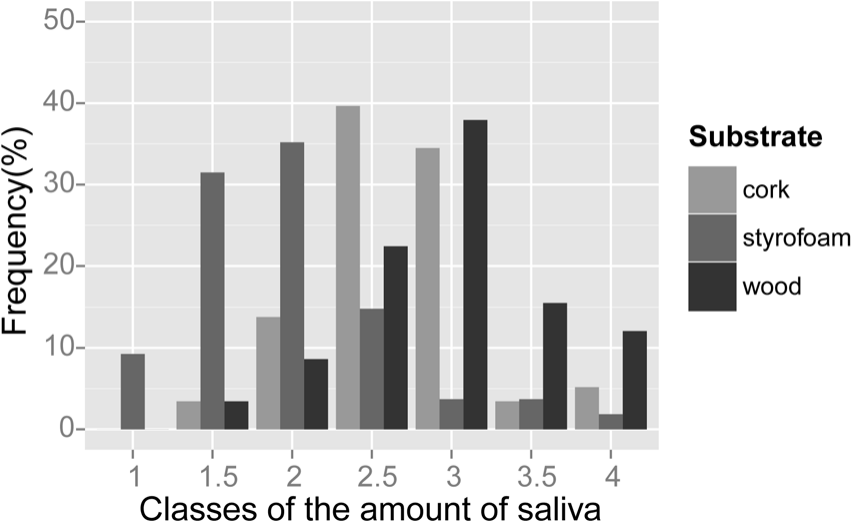
Amount of saliva attached to three types of substrate after dog handling. Bars indicate the distribution of substrates among classes of collected amount of saliva, from absence (class 1) to saturation (class 4). Data from all five experimental sessions are shown. Cork, *n* = 58; styrofoam, *n* = 54; wood, *n* = 58.

No collected substrate was dry and, in general, pieces retained a large quantity of saliva (mean ±SD saliva score: 2.62±0.75). This mean value roughly corresponded to 2–5 ml of saliva. Substrate type greatly influenced the amount of saliva attached to it (*F*_2,131_ = 34.73, *P*<0.001; Fig 2). Mean saliva score for wood was significantly higher than mean scores for cork and styrofoam (Tukey contrasts; *P* = 0.022 and *P*<0.001, respectively), whereas mean score for cork was significantly higher than that for styrofoam (*P*<0.001; Table 2). Differences in mean saliva score for bait types were not statistically significant (Table 2; *t* = 0.456, *P* = 0.649). Univariate analyses showed that other predictors had weak, non-significant effects on saliva scores except chewing time, which had a significant positive effect (*r* = 0.377, *P*<0.001), and licking time, which had a negative effect (*r* = -0.213, *P* = 0.015). We found a rather flat response to handling time (*r* = 0.106, *P* = 0.223) which, however, had a significant negative effect on the residuals of saliva scores against chewing time (*r* = -0.192, *P* = 0.030). Therefore fixed effects retained in the final GLMM model included substrate, chewing time and handling time. The fit of this model was better than the fit of a competing model where handling time was substituted by licking time (ΔAIC = 12.41). Overall, the effect size of dog behaviour was very small as compared with the effect of substrate type (Table 3). This model explained 61% of variance in the saliva index.

**Table 2:** Performance of substrates and baits. The amount of saliva attached to substrates after being handled by dogs, DNA concentration in saliva extracts, identification success, and number of genotyping replicates needed for individual identification are shown (sample size in brackets). Individual identification is expressed as the proportion of dogs matching all ten loci with the reference genotype (either hair genotype or consensus genotype) and as the percentage of correct loci per dog. Values represent means ± SE (upper half of the table) and proportions (lower half).

	Substrate			Bait	
	Styrofoam (42)	Cork (46)	Wood (46)	Meat (69)	Sardines (65)
Saliva score	2.00 ± 0.11	2.73 ± 0.08	3.07 ± 0.09	2.00 ± 0.11	2.00 ± 0.11
[DNA] (ng μl^-1^)	9.13 ± 1.92	8.22 ± 1.50	6.45 ± 1.21	10.55 ± 1.38	5.02 ± 0.98
Individual ID_hair (% correct loci)	90.9 ± 3.4	78.0 ± 5.4	82.2 ± 4.4	83.3 ± 3.7	83.7 ± 3.8
Individual ID_consensus (% correct loci)	91.0 ± 3.4	78.3 ± 5.3	82.6 ± 4.3	83.0 ± 3.7	84.5 ± 3.6
Number of replicates	1.86 ± 0.21	2.24 ± 0.20	2.41 ± 0.20	2.17 ± 0.16	2.18 ± 0.17
Species ID	0.88	0.87	0.80	0.81	0.89
Individual ID_hair	0.79	0.63	0.61	0.67	0.68
Individual ID_consensus	0.81	0.63	0.65	0.67	0.72
Genotyping errors					
Allelic dropout	0.019	0.014	0.017	0.008	0.023
False alleles	0.014	0.009	0.015	0.014	0.011

**Table 3:** Effects included in minimal adequate Generalized Linear Mixed Models. Parameter estimates of fixed effects influencing the quantity of saliva attached to substrates, DNA concentration in saliva extracts (ng µl^-1^), and dog identification success expressed as a perfect match for 10 loci are shown. Levels styrofoam (substrate), meat (bait), female (sex), and small (dog size) are included in the intercept. All models contain the random effects of dog identity nested within experimental session. Errors: normal (saliva score, DNA concentration), binomial (species and individual identification) and Poisson (number of genotyping attempts needed for a correct identification). S*B: substrate-bait interaction.

	Effect	Estimate	SE	*χ* ^*2*^	*P*
Saliva score	Intercept	2.167	0.118		
	Substrate: cork	0.700	0.104	82.30	<0.001
	Substrate: wood	0.991	0.113		
	Chewing time (s)	0.010	0.003	14.20	<0.001
	Handling time (s)	-0.006	0.002	10.10	0.002
log [DNA]	Intercept	2.719	0.303		
	Bait: sardines	-1.097	0.313	29.03	<0.001
	S*B: cork/sardines	0.333	0.298	11.57	0.021
	S*B: cork/meat	-0.533	0.303		
	S*B: wood/sardines	-0.491	0.297		
	S*B: wood/meat	-0.146	0.297		
	Licking time (s)	-0.019	0.004	22.38	<0.001
Species ID	Intercept	2.015	0.536		
	Sex: male	1.312	0.556	4.90	0.018
	Size: large	-1.466	0.662	5.58	0.027
Individual ID_hair	Intercept	-0.959	1.028		
	Substrate: cork	-2.072	0.742	11.84	0.003
	Substrate: wood	-3.183	0.927		
	Chewing time (s)	0.047	0.017	7.74	0.005
	Saliva score	1.042	0.500	4.34	0.037
Individual ID_consensus	Intercept	0.133	0.652		
	Substrate: cork	-2.850	0.365	61.67	<0.001
	Substrate: wood	-3.054	0.445		
	Chewing time (s)	0.037	0.008	21.14	<0.001
	Saliva score	1.478	0.278	28.18	<0.001
Number of replicates	Intercept	1.271	0.233		
	Substrate: cork	0.450	0.172	11.45	0.003
	Substrate: wood	0.626	0.186		
	Saliva score	-0.345	0.107	10.43	0.001

### DNA Concentration

Mean (±SD) DNA concentration in saliva extracts was 14.0 ± 34.4 ng μl^-1^, with a large variation (range 0.03–289.04 ng μl^-1^). Seventy-five percent of values were <10 ng μl^-1^, and 21% fell in the range 10–60 ng μl^-1^. These percentages were similar across substrate types. The remaining 4% were very large values (66–84 ng μl^-1^ in three styrofoam samples covered with meat, and 185–289 ng μl^-1^ in three cork or wood substrates covered with sardines). We considered these six large values as outliers that were excluded in subsequent analyses then we normalized the variable by taking logarithms.

**Fig 3:**
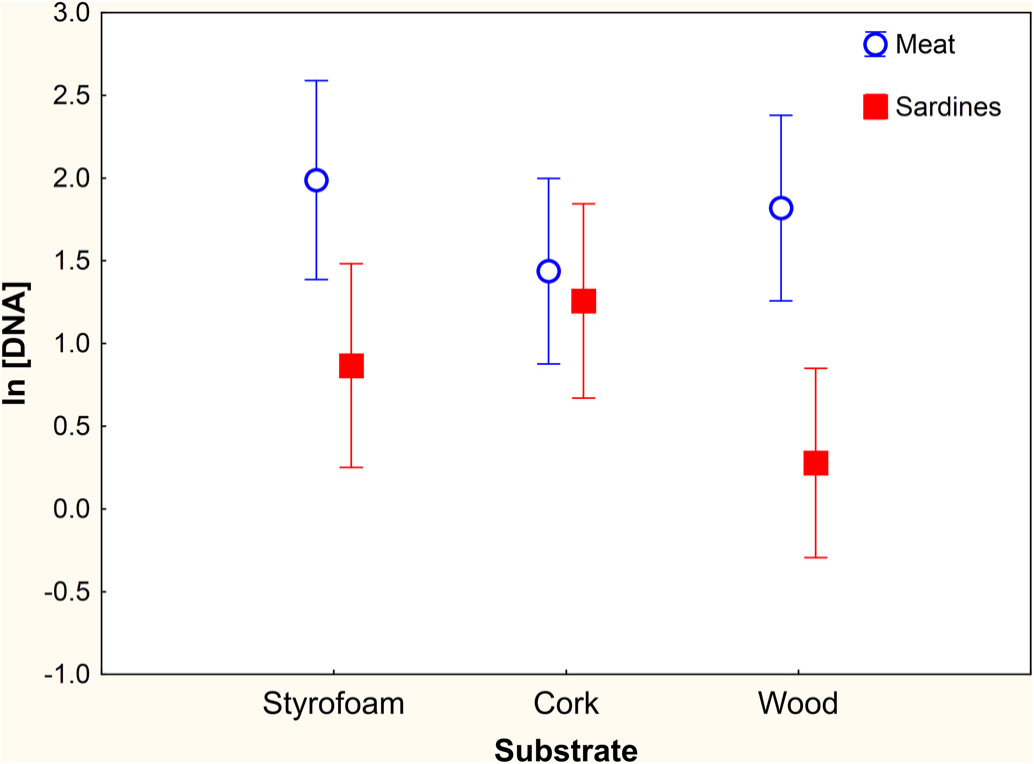
Log-transformed DNA concentration (ng μl^-1^) in saliva extracts. Means and 95% CI are shown for every combination of substrate and bait, once records >60 ng μl^-1^ were excluded.

DNA concentration in extracts was not correlated with the amount of saliva collected in substrates (*r* = 0.118, *P* = 0.184). However DNA concentration greatly varied between types of bait. Mean concentration from substrates supplied with meat was significantly higher (*F*_1,122_ = 15.52, *P*<0.001) than in those with sardines (Table 2). Mean DNA concentration did not vary significantly across substrates (Table 2) but the interaction term was significant, as only wood substrates wrapped with sardines yielded a concentration significantly lower than any kind of substrate covered with meat (Fig 3). Among covariates, licking time had a negative significant effect on DNA concentration. The minimal adequate GLMM contained the effects of bait, the interaction substrate*bait, and licking time (Table 3). Again, the effect size of handling behaviour was small as compared with that of other fixed effects (Table 3). The final model explained 57% of variance in the log-transformed concentration of DNA in saliva extracts.

### Species Identification

**Fig 4:**
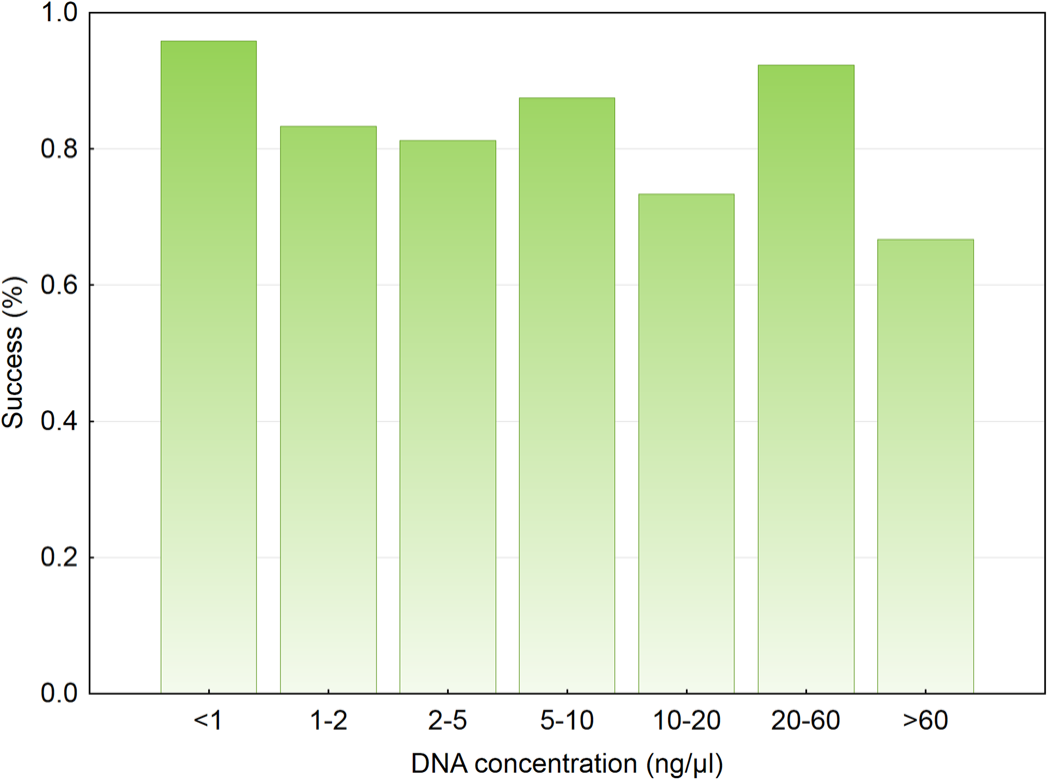
Species identification success across different classes of DNA concentration in saliva extracts.

The mitochondrial marker was amplified in 85.1% of samples and high frequencies of successful identification were little influenced by most predictors. Species identification success was similar across classes of DNA concentration (Fig 4). Success was similarly high in all substrates (Table 2; Wald test = 1.192, *P* = 0.551) and baits (Table 2; Wald test = 1.679, *P* = 0.195). Consequently, the final mixed model included no variable of interest, but contained sex and body mass as significant predictors (Table 3). Success in the identification of males (0.89, *n* = 87) was significantly higher (Wald test = 5.584, *P* = 0.018) than that of females (0.79, *n* = 47), whereas success in small dogs (0.91, *n* = 46) was significantly higher (Wald test = 4.900, *P* = 0.027) than in large dogs (0.82, *n* = 88). The final model explained 7% of deviance in species identification success.

### Individual Identification

Using reference profiles obtained from hair DNA, the percentage of successful individual identification was 67.2%. Samples from session 2 performed poorly (37.1% success) regarding identification success in the other sessions (62.1–88.9%; overall success 77.8%, *n* = 99), and these differences were significant (Wald test = 21.339, *P*<0.001). Our measure of identification success was conservative as it was possible to correctly identify individuals using any subsample of eight loci, for which the percentage of successful individual identification increased to 78%, and up to 90% when the anomalous session 2 was excluded.

**Fig 5:**
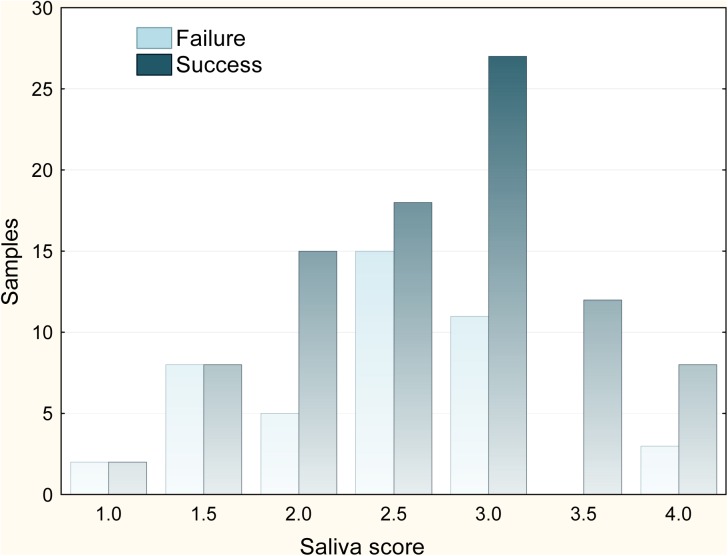
Frequencies of dog individual identification across classes of the amount of saliva attached to the substrate. Successful identification means complete agreement with 10-loci genotypes obtained from hair samples.

**Fig 6:**
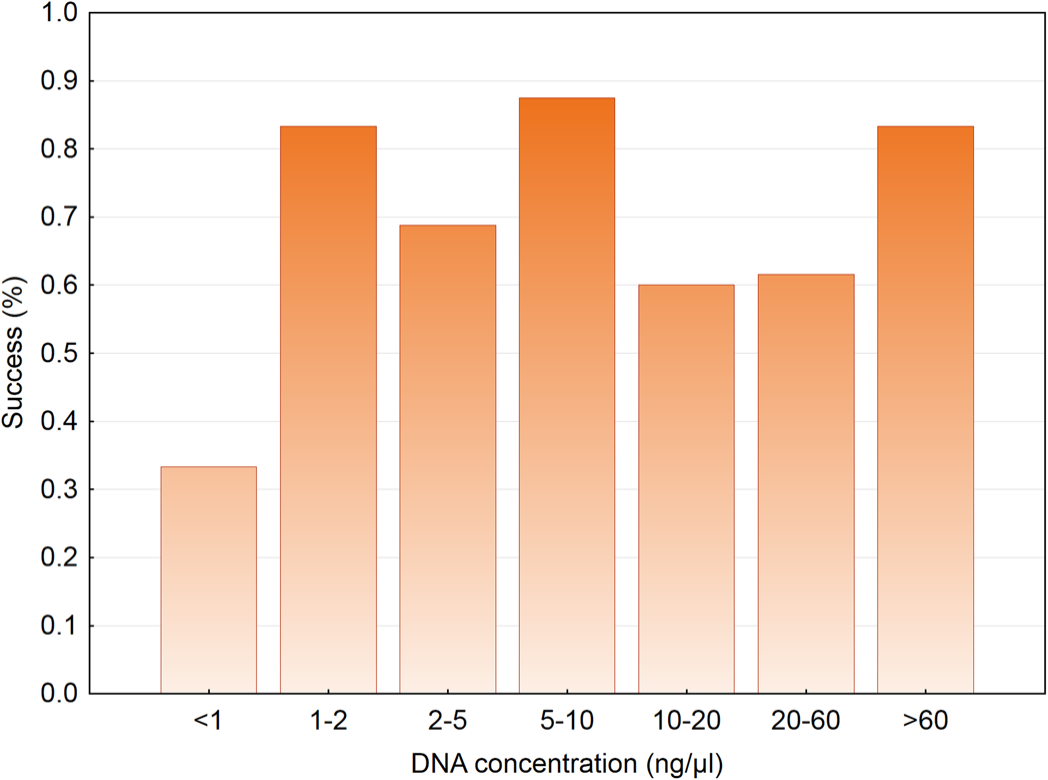
Individual identification success across different classes of DNA concentration in saliva extracts.

The frequency of individual identification increased with the amount of saliva left in substrates (Fig 5; Wald test = 4.361, *P* = 0.037). The frequency of individual identification also increased with the concentration of DNA in saliva extracts. This effect primarily reflected that at DNA concentrations <1 ng μl^-1^ success was less than half that at higher concentrations (*χ* ^*2*^ = 21.162, *df* = 6, *P* = 0.002; Fig 6). Among 24 samples for which DNA concentration was <1 ng μl^-1^, the proportion exposed to sardine baits (0.75) significantly departed from an expected value of 0.5 (goodness of fit *χ* ^*2*^_c_ = 5.917, *df* = 1, *P* = 0.015). Individual identification success for styrofoam was higher than for other substrates, whereas similar values were observed for both bait types (Table 2). The final mixed model retained the significant effects of substrate, amount of saliva and the positive effect of chewing time (Table 3). The effect size of chewing time was much lower than that of other predictors (Table 3).

Genotyping errors were very small across all conditions (allelic dropout <2.3%; false alleles <1.6%; Table 2). When defined as a perfect match for all loci, individual identification success was very similar regardless the type of genotype used as a reference: out of 90 correct identifications according to reference profiles obtained from hair DNA, 99% were also considered correct using consensus genotypes as a reference; likewise, out of 44 unsuccessful identifications established by hair DNA, 91% were deemed incorrect when consensus genotypes were the reference. As a result, using reference consensus genotypes the percentage of successful individual identification even was slightly higher (67.4%) than that reported for hair DNA, and the final model contained the same predictors and effects (Tables 2 and 3). We obtained identical qualitative results when individual identification success was expressed as the proportion of loci correctly identified using either genotypes from DNA hair extracts or consensus genotypes from saliva extracts as a reference (Table 4).

**Table 4:** Minimal adequate binomial GLMMs for dog identification success expressed as the percentage of correct loci. Parameter estimates of fixed effects are shown. For the effects of substrate, bait, dog sex and dog size the levels styrofoam, meat, female, and small, respectively, are included in the intercept. All models contain the random effects of dog identity nested within experimental session.

	Effect	Estimate	SE	*χ* ^*2*^	*P*
Individual ID_hair (% correct loci)	Intercept	0.143	0.641		
	Substrate: cork	-2.909	0.370	62.85	<0.001
	Substrate: wood	-3.142	0.451		
	Chewing time (s)	0.035	0.008	18.71	<0.001
	Saliva score	1.492	0.278	28.86	<0.001
Individual ID_consensus (% correct loci)	Intercept	0.133	0.652		
	Substrate: cork	-2.850	0.365	61.67	<0.001
	Substrate: wood	-3.054	0.445		
	Chewing time (s)	0.037	0.008	21.14	<0.001
	Saliva score	1.478	0.278	28.18	<0.001

Mean number of genotyping replicates that allowed a correct matching for all 10 loci was 2.18 (SD = 1.36). Mean genotyping effort was lowest in styrofoam substrates and highest in wood substrates, but it was similar across baits (Table 2). According to the final mixed model, differences in effort were significant across all substrate levels, and the number of replicates needed for individual identification was inversely proportional to the amount of saliva collected (Table 3).

## Discussion

All substrates had saliva in an amount appreciable to the naked eye. The observed mean sample score (>2.5) roughly corresponded to 2 ml of saliva, which was of the same order of magnitude than volumes obtained through invasive buccal swabbing using pieces of cotton rope of size similar to that of substrates we employed [43,44]. Chemical stimulation of saliva secretion [45] was not needed as edible baits served this function. We were uncertain about the speed of saliva attachment to substrates. A thin layer of saliva might suffice to cover the surface and penetrate into the pores or, alternatively, longer exposure may be needed for viscous saliva to enter pores through capillarity. We collected substrates soon after saliva deposition, i.e. 1-min exposure to dog handling followed by an additional 1-min period allowing further saliva penetration into substrate pores, and obtained enough DNA to identify most individuals. Therefore, the conditions of our experiment support the hypothesis of a quick and effective saliva trapping. The fraction of saliva not retained by substrate surface or pores could have been detached, then solved or suspended in ethanol during storage, and eventually discarded prior to DNA extraction. The quantity of saliva actually available for DNA extraction could thus be independent of the amount of saliva covering substrates at collection, which was supported by the lack of relationship between saliva score and DNA concentration.

Mean DNA concentration in saliva extracts was similar to values obtained from invasive sampling in non-mammal vertebrates (skin swabs in amphibians [46]; plucked feathers and egg swabs in birds [47,48]) or noninvasive sampling in wild canids (faeces [49]; urine [50]). However, the range of DNA concentration values in our study was well above those reported from noninvasive sampling in other mammals (hairs [51,52]; faeces [53,54]). Individual identification success was independent of DNA concentration provided this was >1 ng μl^-1^, otherwise success halved. DNA concentration values may overestimate the amount of dog DNA actually available in saliva extracts for two reasons. First, some DNA could come from the bait itself. Second, dog saliva contains a diversity of bacteria, fungi and other microbiota [55,56]. A substantial fraction of extracted DNA may not belong to the sampled dog because microorganism density in saliva may be even higher than host cell density, as it has been reported in humans [57]. Together with DNA oxidation, concentration of dog DNA in saliva below the 1 ng μl^-1^ threshold could limit amplification and could help decrease individual identification success.

Species identification success (85%) was similar to that reported from other noninvasive sources in canids (79%-94% for faeces [17,58–61]; 55%-87% for hairs [59,60]; 83% for urine [62] but much higher than published figures from saliva samples in prey remains (13–54% [30,63], suggesting that early collection of saliva after deposition may increase the chances of species identification. We found little variation in species identification success, suggesting that the probability of species identification might remain high under a variety of combinations of substrates, baits, volume of saliva collected and DNA concentration in extracts. We cannot advance a convincing explanation for the effects of covariates (sex and size) retained in the final model which, after all, had very low explanatory power.

Individual identification success was conservatively estimated as 67% but, once the anomalous results of session 2 were removed and success computed on eight microsatellite markers, our method produced a remarkable success of 90%. Again, this value falls close to the upper end of the range of values reported for noninvasive sampling in canids (53–96% [17,58,62,64–67]). We achieved individual identification using on average two replicates, an effort definitely lower than that recommended [68] or usually employed when using noninvasive samples (3–8 replicates [12,60,69]). Reduced replication saves laboratory costs and delays exhaustion of extracted DNA [52,59]. Finally, our method was accurate as suggested by remarkably low genotyping error rates. As expected from good quality DNA in a freshly collected secretion, mean allelic dropout (<2%) was much smaller than values reported for more aged noninvasive samples in canids (18–69% [30,58,62].

High genotyping success could be attributed to cell richness in saliva. In humans the mean density of nucleated cells present in saliva, mostly epithelial cells exhibiting high turnover [57], is as high as in blood [70]. Other factors affecting individual identification success (chewing time, amount of saliva collected, and substrate type) remained the same when success was obtained either from reference genotypes or consensus genotypes. Consistently, the number of replicates needed for individual identification was essentially affected by the same factors. As expected, the amount of saliva collected was positively associated with how long dogs handled substrates inside their mouths. The additional negative effect of handling time suggests that part of that saliva was lost through contact with the dog’s body or surrounding objects. The probability of mechanical removal of saliva could increase with handling duration once bait has been ingested, especially when long licking times follow lengthy chewing periods. Licking during this phase might add some saliva but could equally remove saliva previously deposited on the substrates, especially when they were held against the ground for licking. The negative effect of handling time suggests that the proportion of saliva detached by friction is proportional not only to licking time but also to the amount of saliva deposited (expressed by chewing time), which is a plausible explanation considering the viscous nature of dog saliva.

We designed this pilot study under controlled conditions in order to estimate the potential performance of this technique for ecological applications. Our analyses revealed further practical aspects that should be borne in mind and refined when designing field studies.

Despite their lower volume, wood substrates collected a larger amount of saliva but tended to yield a lower DNA concentration than other materials. Wood might absorb less saliva, or might absorb it more slowly, than cork and styrofoam if, as we perceived, wood porosity were comparatively low. Reduced absorption would keep a larger volume of saliva on the wood surface that may be lost after immersion in ethanol. Higher DNA concentrations could have been obtained by collecting precipitates after ethanol evaporation. This excess of saliva is unlikely to be found in the field as in most cases time elapsed between deposition and collection may allow substrates to dry out, but saliva cells may remain on their surface.

We noted that organic substrates performed worse than styrofoam in terms of individual identification, probably because the presence of phenolic components cause oxidative DNA breakage [71,72] and also inhibit the PCR [73]. As polyphenols abound in wood and cork [74,75] but are supposedly absent in synthetic polymers, differences in individual identification rates across substrates might be related with reduced amplification because of DNA degradation rather than to scarcity of DNA molecules. Styrofoam might be free of organic PCR inhibitors, but it is also a soft material that can be broken, removed or even swallowed, which may entail drawbacks for both saliva collection and animal welfare. Polyphenols also occur in some oils used in the fish processing industry [76]. Oils in canned sardines may have covered the surface of substrates exposing DNA molecules to further degradation, which is consistent with the negative effect of sardine baits on DNA concentration. This effect was especially marked in the wood-sardine combination, whereas in more porous materials (cork and styrofoam) oils might not cover all microsurfaces and a fraction of DNA might be protected from further degradation by polyphenols. As sardine baits were overrepresented in samples with DNA concentration <1 ng μl^-1^, low rate of individual identification in these samples may be partly due to pro-oxidants catalyzing DNA degradation. Finally, the second session of our experiment had to be carried out in pens with soil, plant litter and other organic matter. Poor results in individual identification for this batch of saliva samples could also be attributed to contamination with PCR inhibitors present in the ground [73].

In general, technical improvements for field applications should include ways to i) design bait stations that quickly attract and reward elusive species: animal wariness may delay a regular use of feeding devices [77] which may decrease the efficiency and increase the costs of saliva collection, but this issue can be partly mitigated by using techniques that elicit the investigative behaviour of animals [78,79]; ii) maximize duration of substrate inside the animal’s mouth to collect as much saliva as possible: substrates would be better presented attached to structures favouring extended handling periods while impeding substrate removal (e.g. through tethering [80]); iii) minimize saliva contact with different sources of PCR inhibitors, present both in and around the substrate: both substrates and baits should be inhibitor-free when possible, and contact with the ground should be prevented, for example, by anchoring substrates to vertical structures or suspending them from tree branches, poles or stakes [81,82]; iv) minimize saliva loss to attain high individual identification rates: soft or fragile materials can be swallowed or removed and should be avoided, whereas porous substrates would be preferred because pores might protect DNA in saliva cells from UV radiation [83] and saliva trapped inside pores might have a lower probability to be washed away by the rain; and v) preventively minimize the time elapsed between deposition and collection, as DNA degradation in noninvasive samples is generally expected to increase over time [11,12]; for example, collection of saliva samples can be combined with monitoring methods that require daily checking such as scent stations [84]. The effect of age of saliva samples on the rate of genotyping success deserves additional study.

The substrate-bait method of saliva collection proved efficient for individual identification under controlled conditions. Further experiments are needed to develop ways of adapting this technique to specific field settings for the ecological study of elusive animal targets. Provided that these practicalities are resolved, our results offer promising prospects for saliva use as a source of DNA in noninvasive surveys of terrestrial vertebrates. Specifically, the substrate-bait method i) uses simple and inexpensive saliva traps that are easily prepared and transported, ii) has the potential to provide high species and individual identification rates, iii) may reduce lab time and costs because potentially high average DNA concentration would allow PCR replication to be kept to a minimum, and iv) can be used as an alternative or complement to more expensive monitoring techniques such as camera trapping [85].

## Supporting Information

S1 Table Ten dog autosomal microsatellite used for individual identification. (PDF)
