# Supplementary material for: A New Method for Noninvasive Genetic Sampling of Saliva in Ecological Research
Source: PLoS One. 2015 Oct 23;10(10):e0139765. doi: 10.1371/journal.pone.0139765 (PMC4619700; doi:10.1371/journal.pone.0139765)
Supplement: S1 Table — (PDF) [file pone.0139765.s001.pdf]

**S1 Table.** Ten dog autosomal microsatellite used for individual identification. Repeat type (di- or tetra-nucleotide), primer concentration, and fluorescent dye are shown

| Locus | Repeat | Concentration (pM/µl) | Dye | Source |
|---|---|---|---|---|
| C08.410 | di | 0.20 | VIC | Moore *et al.* (2010) |
| C08.618 | di | 0.16 | VIC | Moore *et al.* (2010) |
| C09.474 | di | 0.28 | PET | Neff *et al*. (1999) |
| C22.763 | di | 0.36 | PET | Oberbauer *et al*. (2003) |
| CPH5 | di | 0.16 | FAM | Fredholm and Wintero (1995) |
| CPH2 | di | 0.20 | NED | Fredholm and Wintero (1995) |
| CPH9 | di | 0.16 | NED | Fredholm and Wintero (1995) |
| CXX.459 | di | 0.16 | VIC | Ostrander *et al.* (1995) |
| FH2161 | tetra | 0.28 | NED | Francisco *et al.* (1996) |
| REN64E19 | di | 0.16 | FAM | Breen *et al*. (2001) |

**References**

Breen M, Jouquand S, Renier C , Mellersh CS, Hitte C, Holmes NG, Cheron A, Suter N, Vignaux F, Bristow AE, Priat C, McCann E, André C, Boundy S, Gitsham P, Thomas R, Bridge WL, Spriggs HF, Ryder EJ, Curson A, Sampson J, Ostrander EA, Binns MM, Galibert F (2001) Chromosome-specific single-locus FISH probes allow anchorage of an 1800-marker integrated radiation-hybrid/linkage map of the domestic dog genome to all chromosomes. Genome Res 11:1784-1795

Francisco LV, Langston AA, Mellersh CS, Neal CL, Ostrander EA (1996) A class of highly polymorphic tetranucleotide repeats for canine genetic mapping. Mamm Genome 7:359-362

Fredholm M, Wintero AK (1995) Variation of short tandem repeats within and between species belonging to the *Canidae* family. Mamm Genome 6:11-18

Moore M, Brown SK, Sacks BN (2010) *Vulpes vulpes* In: Molecular Ecology Resources Primer Development Consortium, Permanent Genetic Resources added to Molecular Ecology Resources Database 1 October 2009–30 November 2009. Mol Ecol Resour 10:404-408

Neff MW, Broman KW, Mellersh CS, Ray K, Acland GM, Aguirre GD, Ziegle JS, Ostrander EA, Rine J (1999) A second-generation genetic linkage map of the domestic dog, *Canis familiaris*. Genetics 151:803-820

Oberbauer AM, Grossman DI, Irion DN, Schaffer AL, Eggleston ML, Famula TR (2003) The genetics of epilepsy in the Belgian tervuren and sheepdog. J Hered 94:57-63

Ostrander EA, Mapa FA, Yee M, Rine J (1995) 101 new simple sequence repeat-based markers for the canine genome. Mammal Genome 6:192-195
